# Activation of a Mitogen-Activated Protein Kinase Hog1 by DNA Damaging Agent Methyl Methanesulfonate in Yeast

**DOI:** 10.3389/fmolb.2020.581095

**Published:** 2020-12-11

**Authors:** Shan Huang, David Zhang, Fangli Weng, Yuqi Wang

**Affiliations:** Department of Biology, Saint Louis University, St. Louis, MO, United States

**Keywords:** Hog1, DNA damage, mitogen-activated protein kinase, Ypd1, autophagy, yeast Hog1 activation by DNA damage

## Abstract

Hog1 is a mitogen-activated protein kinase in yeast that primarily regulates cellular responses to hyperosmolarity stress. In this study, we have examined the potential involvement of Hog1 in mediating cellular responses to DNA damaging agents. We find that treatment of yeast cells with DNA damaging agent methyl methanesulfonate (MMS) induces a marked and prolonged Hog1 activation. Distinct from stressors such as arsenite that activates Hog1 via inhibiting its phosphatases, activation of Hog1 by MMS is phosphatase-independent. Instead, MMS impairs a critical phosphor-relay process that normally keeps Hog1 in an inactive state. Functionally, MMS-activated Hog1 is not translocated to the nucleus to regulate gene expression but rather stays in the cytoplasm and regulates MMS-induced autophagy and cell adaptation to MMS stress. These findings reveal a new role of Hog1 in regulating MMS-induced cellular stress.

## Introduction

Mitogen-activated protein kinases (MAPKs) are key components of cellular signaling pathways that allow eukaryotic cells to respond to a broad range of environmental signals. Hog1 is a MAPK in the budding yeast *Saccharomyces cerevisiae*, and its well-established role is regulating cellular responses to hyperosmotic stress (Saito and Posas, 2012). Accumulating evidence indicates that Hog1 has a much broader role and can respond to many other cellular stresses, which include heat shock (Winkler et al., 2002), citric and acetic acid (Lawrence et al., 2004; Mollapour and Piper, 2006), cold shock (Panadero et al., 2006), oxidative stress (Bilsland et al., 2004), methylglyoxal (Aguilera et al., 2005), bacterial lipopolysaccharide (Marques et al., 2006), glucose starvation (Piao et al., 2012), curcumin (Azad et al., 2014), cadmium (Jiang et al., 2014), and arsenite (Sotelo and Rodriguez-Gabriel, 2006; Thorsen et al., 2006). Among these, activation of Hog1 by arsenite is very noteworthy, because it is achieved via a direct inhibition of the phosphatases Ptp2 and Ptp3 (Lee and Levin, 2018), establishing a precedence of MAPK activation that is independent of the upstream components.

One type of stressor that a living organism continuously experiences throughout its lifetime is DNA damaging agents, which can arise from different sources in the environment that include ultraviolet rays, ionizing radiation, toxic cellular metabolism byproducts, and genotoxic chemicals (Mehta and Haber, 2014; Chatterjee and Walker, 2017). Cells can elicit a variety of DNA damage responses to repair and restore the genetic information to avoid an accumulation of deleterious mutations and chromosomal aberrations (Chatterjee and Walker, 2017). One of the responses is autophagy, a catabolic process that removes a portion of cytoplasm for lysosomal degradation and recycling (Eapen et al., 2017; Galati et al., 2019). It is believed that at a low level of DNA damage, augmented autophagy helps to promote survival by providing energy to sustain the need for DNA repair, while at a high level of DNA damage, enhanced autophagy promotes cell death and thus facilitates the removal of severely damaged cells (Galati et al., 2019).

In yeast, it has been shown that DNA damaging agents such as methyl methanesulfonate can activate Mpk1/Slt2, a MAP kinase primarily responsible for cell wall maintenance (Soriano-Carot et al., 2012; Liu and Levin, 2018; Lee et al., 2019). Interestingly, activation of Mpk1/Slt2 by methyl methanesulfonate is achieved via induced proteasomal degradation of Msg5, a phosphatase that helps to maintain Mpk1/Slt2 at a low basal level of activation (Liu and Levin, 2018). Whether Hog1 is similarly activated by DNA damaging agents is unknown. Given that the basal level of Hog1 is also maintained by its phosphatases (Lee and Levin, 2018), we became interested in if DNA damage could also induce Hog1 activation by enhancing the degradation of its phosphatases. In addition, Hog1 is known to be involved in the regulation of autophagy (Prick et al., 2006; Mao et al., 2011), raising the possibility that Hog1may be activated by DNA damage to regulate the extent of autophagy. In this study, we investigated these possibilities by testing the effect of DNA damaging agent methyl methanesulfonate (MMS) on Hog1. Our results indicate that MMS does lead to Hog1 activation and that Hog1 is required for the full induction of MMS-triggered autophagy. Furthermore, we find that the Sln1 branch of the HOG pathway is primarily responsible for mediating MMS-induced Hog1 activation.

## Results

### MMS Induces Activation of Hog1

**Figure 1:**
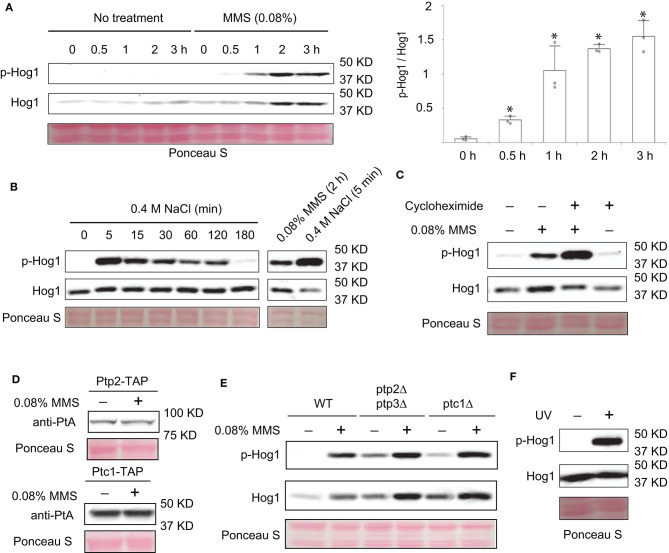
MMS treatment leads to activation of Hog1. **(A)** Wild type cells were grown to mid-log phase and treated or not treated with 0.08% MMS for the indicated time. Whole cell extracts were resolved on 8% SDS-PAGE and probed with anti-phospho-p38 (#9212, Cell Signaling Technology) and anti-Hog1 (SC-165978, Santa Cruz). Equal loading was verified with Ponceau S staining. p-Hog1, phosphorylated and activated Hog1. Quantification of immunoblots by densitometry from three independent experiments is shown in the lower panel. The difference between each time point and time 0 was statistically analyzed (*p < 0.050). **(B)** Wild type cells in mid-log phase were treated with 0.4 M NaCl for the indicated time. Whole cell extracts were analyzed by western blotting with anti-phospho-p38 and anti-Hog1. A direct comparison between cells treated with 0.08% MMS for 2 h and with 0.4 M NaCl for 5 min was shown on the right panel. **(C)** Wild type cells in mid-log phase were treated with 0.08% MMS, 30 μM cycloheximide, or both for 2 h. Whole cell extracts were analyzed by western blotting with anti-phospho-p38 and anti-Hog1. **(D)** Cells with TAP-tag on the genomic locus of *PTP2* and *PTC1* were grown to mid-log phase and treated or not treated with 0.08% of MMS for 2 h. Whole cell extracts were resolved on 8% SDS-PAGE and probed with anti-protein A (anti-PtA, P2921, Sigma-Aldrich). Equal loading was verified with Ponceau S staining. **(E)** Wild type cells or *ptp2*Δ*ptp3*Δ or *ptc1*Δ mutants were grown to mid-log phase and treated or not treated with 0.08% of MMS for the indicated time. Whole cell extracts were resolved on 8% SDS-PAGE and probed with anti-phospho-p38 (#9212, Cell Signaling Technology) and anti-Hog1 (SC-165978, Santa Cruz). Equal loading was verified with Ponceau S staining. p-Hog1, phosphorylated and activated Hog1. The data shown are representative of three independent experiments. **(F)** Wild type cells in mid-log phase were treated or not treated with ultraviolet irradiation for 2 min. Whole cell extracts were analyzed by western blotting using anti-phospho-p38 and anti-Hog1.

Hog1and Slt2/Mpk1 are two yeast MAPKs that are important for stress responses. It has been shown that Slt2/Mpk1 can be activated by DNA damaging agents such as MMS and hydroxyurea (Liu and Levin, 2018). However, whether Hog1 can be similarly activated is unknown. To test this, we grew yeast cells to mid-log phase, treated or not treated the cells with 0.08% of MMS (Liu and Levin, 2018), and monitored the activation level of Hog1, using an antibody that recognizes the doubly-phosphorylated and thus activated Hog1. As shown in Figure 1A, MMS treatment led to a clear activation of Hog1, which can be detected as early as 30 min after the treatment. Distinct from high osmolarity-induced Hog1 activation, which is transient and occurs quickly (Figure 1B) (English et al., 2015), MMS induced a prolonged Hog1 activation, which persisted even 3 h post-treatment (Figure 1A). The level of MMS-induced Hog1 activation is clearly less than that induced by high osmolarity (Figure 1B). In addition to phosphorylated Hog1, the level of Hog1 protein was also clearly increased upon MMS treatment, especially in the later time points (2 and 3 h post-treatment). To examine if new protein synthesis of Hog1 protein induced by long term MMS treatment is responsible for the observed increase in phosphorylated Hog1, we examined the effect of MMS on Hog1 activation with or without the addition of protein synthesis inhibitor cycloheximide. As shown in Figure 1C, cycloheximide did prevent MMS-induced increase in the level of Hog1 protein but not that of phosphorylated Hog1, indicating the observed increase in Hog1 phosphorylation is not due to an elevated level of Hog1 protein. Notably, co-treatment of cells with MMS and cycloheximide led to an even higher level of Hog1 phosphorylation, suggesting the existence of a new protein synthesis-dependent mechanism that limits the extent of MMS-induced Hog1 activation.

Next, we sought to determine if phosphatases are involved in MMS-induced activation of Hog1. We considered this possibility because there is precedence for MAPK activation caused by elevated degradation of its phosphatase (Liu and Levin, 2018). It is possible that MMS treatment can also induce degradation of Hog1-specific phosphatases, which in turn leads to an increase in Hog1 activation. To test this, first we examined if MMS treatment affects the abundance of Ptp2 and Ptc1, two phosphatases known to inactivate Hog1 (Wurgler-Murphy et al., 1997; Warmka et al., 2001). As shown in Figure 1D, the abundance of neither of these proteins was diminished upon MMS treatment. In addition, we also examined the behaviors of mutants that lack these phosphatases. As shown in Figure 1E, the phosphatase mutants (*ptp2*Δ*ptp3*Δ, and *ptc1*Δ) displayed a higher basal level of phosphorylated Hog1. However, an elevated Hog1 phosphorylation still occurred in these mutants upon MMS treatment, indicating that MMS-induced Hog1 activation is independent of its phosphatases Ptp2, Ptp3, and Ptc1.

MMS is a genotoxic chemical that causes DNA damage by alkylating bases including guanine and adenine (Beranek, 1990). To check if Hog1 activation can be induced by other types of genotoxic stressors, we subjected cells to the treatment of ultraviolet radiation and examined the effect on the level of Hog1 phosphorylation. As shown in Figure 1F, ultraviolet radiation treatment clearly led to increased Hog1 phosphorylation, suggesting Hog1 can be activated by other DNA damaging agent and not just by MMS specifically.

### MMS-Induced Hog1 Activation Is Mediated by the Sln1 Branch of the HOG Pathway

**Figure 2:**
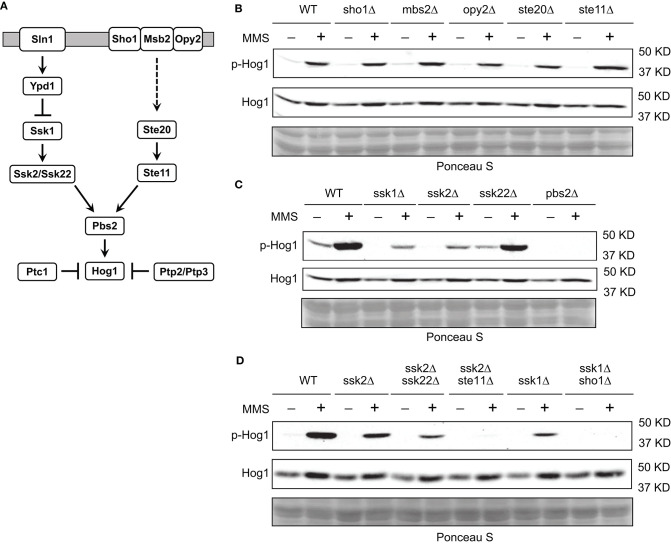
Sln1 branch of the HOG pathway is mainly responsible for MMS-induced Hog1 activation. **(A)** The HOG pathway. See text for details. **(B)** Wild type cells or isogenic mutants lacking individual components in the Sho1 branch of the HOG pathway were grown to mid-log phase and treated or not treated with 0.08% of MMS for 2 h. Whole cell extracts were resolved on 8% SDS-PAGE and probed with anti-phospho-p38 (#9212, Cell Signaling Technology) and anti-Hog1 (SC-165978, Santa Cruz). Equal loading was verified with Ponceau S staining. p-Hog1, phosphorylated and activated Hog1. The data shown are representative of three independent experiments. **(C)** The same experiments were carried out as in panel **(B)** for mutants lacking individual component in the Sln1 branch of the HOG pathway. **(D)** The same experiments were carried out as in panel **(B)** for double deletion mutants.

To better understand how MMS induces Hog1 activation, we reasoned that it would be helpful to identify the upstream components in the HOG pathway that are important for MMS-induced Hog1 activation. To this end, we examined the behaviors of mutants lacking each individual component in the two branched pathways that lead to Hog1 activation (Saito and Posas, 2012) (Figure 2A). We find that disrupting each individual component in the Sho1 branch of the HOG pathway has nearly no effect on MMS-induced Hog1 activation (Figure 2B). However, disrupting either Ssk1 or Ssk2, the two key components in the Sln1 branch of the HOG pathway, substantially diminished the level of MMS-induced Hog1 activation (Figure 2C), indicating that upstream components from the Sln1 branch of the HOG pathway are important for MMS-induced Hog1 activation. The residual Hog1 activation observed in the *ssk1*Δ and *ssk2*Δ mutants may be due to the input from the Sho1 branch and/or redundancy between Ssk2 and Ssk22. To investigate this, we created three double deletion mutants, i.e., *ssk2*Δ *ssk22*Δ, *ssk2*Δ *ste11*Δ, and *ssk1*Δ *sho1*Δ, and examined their behavior. As shown in Figure 2D, the *ssk2*Δ *ssk22*Δ mutant displayed a lower level of MMS-induced Hog1 phosphorylation than the *ssk2*Δ mutant, indicating a redundant role of Ssk2 and Ssk22. In addition, MMS-induced Hog1 phosphorylation was nearly completely gone in both the *ssk2*Δ *ste11*Δ and *ssk1*Δ *sho1*Δ mutants, suggesting the Sho1 branch may be responsible for the residual phosphorylation observed in the *ssk1*Δ and *ssk2*Δ mutants. It is also possible that the residual response observed in the *ssk1*Δ or *ssk2*Δ mutants was the result of the amplification of MMS on the basal signal via some unknown mechanism, and removing the Sho1 branch from the *ssk1*Δ or *ssk2*Δ mutants blocks the basal signal that flows through the pathway to Hog1 and thus the response.

**Figure 3:**
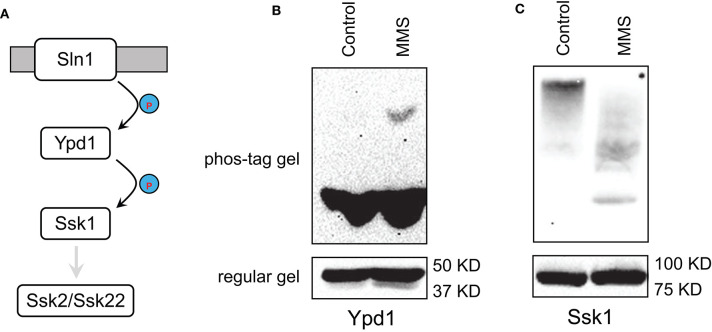
MMS interferes with phosphor-relay in the Sln1/Ypd1/Ssk1 axis. **(A)** The phosphor-relay in the Sln1/Ypd1/Ssk1 axis. Phosphorylation prevents Ssk1 from interacting and activating its downstream Ssk2/Ssk22. **(B)** Cells with TAP-tag on the genomic locus of *YPD1* were grown to mid-log phase and treated or not treated (Control) with 0.08% of MMS for 2 h. Whole cell extracts were resolved on 8% phos-tag (10 μM) containing or regular SDS-PAGE and probed with anti-protein A (P2921, Sigma-Aldrich). **(C)** Cells with TAP-tag on the genomic locus of *SSK1* were grown to mid-log phase and treated or not treated (Control) with 0.08% of MMS for 2 h. Whole cell extracts were resolved on 8% phos-tag (10 μM) containing or regular SDS-PAGE and probed with anti-protein A (P2921, Sigma-Aldrich).

In the Sln1 branch of HOG pathway, Sln1/Ypd1/Ssk1 is a variant of the two-component signaling system, in which Sln1 is a histidine kinase, Ssk1 is a receiver, and Ypd1 is an intermediate that transfers phosphate from Sln1 to Ssk1 to keep Ssk1 inactive (Figure 3A) (Saito and Posas, 2012). Sln1 senses an increase in the osmolarity and becomes inactivated, which in turn leads to diminished phosphor-transfer from Sln1 to Ypd1 to Ssk1, resulting in the pathway activation (Posas et al., 1996). Given the central role of Ypd1 in this phosphor-transfer and pathway activation, we sought to determine if MMS treatment has any impact on Ypd1. Accordingly, we treated cells that express TAP-tagged Ypd1 on its genomic locus with MMS, separated protein extracts on SDS-PAGE that contains phos-tag, and immunoblotted Ypd1-TAP. Phos-tag was used to facilitate the detection of phosphorylated Ypd1 as it retards migration of phosphorylated proteins on SDS-PAGE (Horinouchi et al., 2016; Huang et al., 2019). As shown in Figure 3B, MMS treatment does not alter the abundance of Ypd1 but leads to an accumulation of phosphorylated Ypd1. This finding suggests that phosphor-relay along the Sln1/Ypd1/Ssk1 axis is disrupted by MMS treatment, which in turn could lead to Hog1 activation. This model predicts that in addition to an accumulation of phosphorylated Ypd1, there should be a decrease in the level of phosphorylated Ssk1. To test this, we treated cells that express TAP-tagged Ssk1 on its genomic locus with MMS, separated protein extracts on SDS-PAGE that contains phos-tag, and immunoblotted Ssk1-TAP. As shown in Figure 3C, MMS treatment indeed leads to a clear decrease in the level of phosphorylated Ssk1.

### MMS-Induced Hog1 Activation Regulates Autophagy

**Figure 4:**
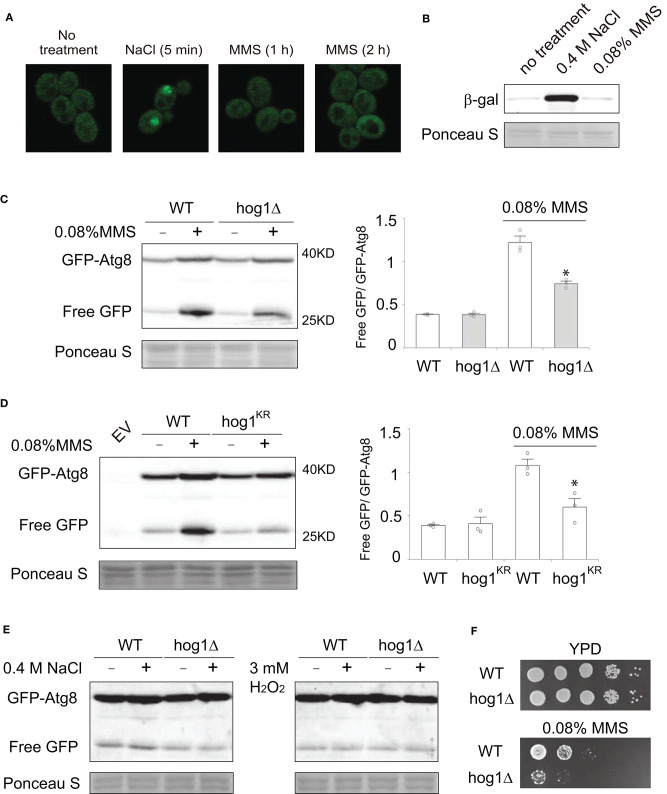
Hog1 is required for MMS-induced autophagy. **(A)** Cells with GFP-tag on the genomics locus of *HOG1* were grown to mid-log phase, treated or not treated with either 0.4 M NaCl (5 min) or 0.08% of MMS for either 1 or 2 h. The localization of GFP-tagged Hog1 was visualized using confocal microscopy. **(B)** Wild type cells transformed with plasmids that express *STL1*-lacZ were grown to mid-log phase, treated with either 0.4 M NaCl or 0.08% MMS for 2 h. Whole cell extracts were resolved on 7% SDS-PAGE and probed with anti-b-galactosidase (Z3781, Promega) and anti-phospho-p38. Equal loading was verified with Ponceau S staining. **(C)** Wild type cells or *hog1*Δ mutants transformed with a plasmid that expresses GFP-Atg8 were grown to mid-log phase, treated or not treated with 0.08% MMS for 2 h. Whole cell extracts were resolved on 10% SDS-PAGE and probed with anti-GFP (ab13970, abcam). Equal loading was verified with Ponceau S staining. Quantification of immunoblots by densitometry from three independent experiments is shown in the lower panel. The difference between wild type and the *hog1*Δ mutants in MMS-treated samples was statistically analyzed (*p < 0.050). **(D)** The same experiments as described in panel **(C)** were carried out except that a catalytic inactive Hog1 mutant, i.e., hog1^K52R^, was used. EV, empty vector. **(E)** The same experiments as described in panel **(C)** were carried out except that 2 h of 0.4 M NaCl treatment was used in the left panel and 2 h of 3 mM H_2_O_2_ treatment was used in the right panel. **(F)** Serial diluted wild type cells or *hog1*Δ mutants were spotted on to either YPD plate or YPD plate containing 0.08% MMS. Plates were incubated at 30°C for 2 days and photographed.

Next, we sought to investigate the physiological relevance of MMS-induced Hog1 activation. To this end, we first examined if MMS treatment impacts subcellular localization of Hog1. It has been well-established that Hog1 has substrates in both the cytoplasm and the nucleus (Saito and Posas, 2012), thus knowledge of its subcellular localization upon activation can be useful in discerning its function. As shown in Figure 4A, upon MMS treatment, the majority of Hog1 is present in the cytoplasm, and there is no clear sign of nuclear accumulation of the protein, whereas salt treatment clearly induces nuclear accumulation of Hog1 as reported previously (Reiser et al., 1999). Likewise, salt treatment induced expression of *STL1*-lacZ, a well-established reporter for Hog1-dependent gene expression (de Nadal et al., 2003), but MMS treatment showed no effect (Figure 4B). These analyses suggest that the physiological process impacted by MMS-induced Hog1 phosphorylation likely occurs in the cytoplasm.

Autophagy is a process in which cells enwrap a portion of the cytoplasm and deliver it to the lysosome for degradation (Parzych and Klionsky, 2014). It plays important roles in regulating the abundance of various proteins involved in DNA repair (Galati et al., 2019). It is possible that MMS-induced activation of Hog1 in the cytoplasm may contribute to the regulation of autophagy. To test this, we examined MMS-induced autophagy in both wild type cells and the *hog1*Δ mutants, using a well-established autophagy reporter GFP-Atg8 (Cheong and Klionsky, 2008). As shown in Figure 4C, MMS treatment induces robust autophagy as judged by the ratio of free GFP and GFP-Atg8. Interestingly, in the *hog1*Δ cells, the extent of autophagy is substantially reduced. To determine if the kinase activity of Hog1 is required for its regulation of MMS-induced autophagy, we tested a catalytically inactive hog1^K52R^ mutant and found that this mutant similarly showed diminished autophagy (Figure 4D). These data indicate that Hog1 is required for the optimal level of MMS-induced autophagy and a role of MMS-induced Hog1 activation is to enhance autophagy. To determine if other stressors that have been reported to activate Hog1 can induce autophagy, we examined hyperosmolarity and hydrogen peroxide. As shown in Figure 4E, neither was able to induce autophagy. Finally, to investigate the physiological significance of MMS-induced Hog1 activation, we compared the MMS sensitivity of wild type and the *hog1*Δ mutant. We found that the *hog1*Δ mutant is more sensitive to MMS treatment than wild type (Figure 4F).

## Discussion

Organisms are constantly under the threat of environmental stressors that could damage their DNA. Understanding molecular mechanisms by which cells respond to DNA damage agents is very important. In this study, we examined the involvement of Hog1, a mitogen-activated protein kinase, in cellular responses to DNA damaging agent MMS. We find that Hog1 becomes activated in responding to MMS treatment. Interestingly, activation of Hog1 is required for the full induction of autophagy triggered by MMS treatment.

It has been demonstrated in the past that certain mitogen-activated protein kinases are involved in DNA damage responses. In mammals, JNK and p38 are known to be activated by DNA damage stressors (Holbrook et al., 1996; Picco and Pages, 2013; Zlotorynski, 2018), but precisely how the DNA damage signal is transmitted to activate these kinases remains unclear (Borisova et al., 2018; Liu and Levin, 2018). In the budding yeast, prior to this work, the only MAPK that has been demonstrated to be activated by genotoxic stress is Mpk1/Slt2 (Dardalhon et al., 2009; Soriano-Carot et al., 2012; Liu and Levin, 2018), a MAPK that is primarily responding to cell wall stress to maintain cell wall integrity (Levin, 2005). Mechanistically, Mpk1/Slt2 is activated by genotoxic stress via accelerated degradation of its phosphatase Msg5 in the proteasome (Liu and Levin, 2018). This is possible because Msg5 and Mpk1/Slt2 form a tight complex and a key role of Msg5 is to keep the Mpk1/Slt2 at low basal level of activation (Liu and Levin, 2018). Our study suggests that genotoxic stress activates Hog1 via a distinct mechanism by impairing phosphorelay between Ypd1 and Ssk1. This model is supported by our genetic analysis, which indicates that the Sln1 branch of the HOG pathway is primarily required for MMS-induced activation of Hog1, as disrupting the components such as Ssk1 and Ssk2 in the Sln1 branch affects MMS-induced Hog1 activation but removing the components such as Ste20 and Ste11 in the Sho1 branch on their own has no effect. An impaired phosphorelay between Ypd1 and Ssk1 is also supported by our biochemical investigation, which revealed a clear accumulation of phosphorylated Ypd1 and an apparent decrease of Ssk1 phosphorylation. The accumulation of phosphorylated Ypd1 upon MMS treatment is consistent with the model that phosphor-transfer from Sln1 to Ypd1 occurs but its further transfer from Ypd1 to Ssk1 is somehow impaired. Given the prevalence of two-component phosphorelay systems in prokaryotes and plants (Pirrung, 1999; Adam and Hunter, 2018), it would be interesting to investigate whether they respond to DNA damage in a similar manner. The canonical two-component signaling system has not been identified in mammals; part of the reason is due to the challenge of studying phosphorylation of histidine and glutamate (Adam and Hunter, 2018). However, histidine kinases do exist in mammals, and one example is NM23, which has been demonstrated to associate with DNA damage sites (Wagner and Vu, 2000), consistent with its possible role in sensing and responding to DNA damage.

It is not clear how DNA damage impairs the phosphorelay between Ypd1 and Ssk1. Notably, Ypd1 is located in both the cytoplasm and the nucleus (Huh et al., 2003). Upon phosphorylation by Sln1, Ypd1 can in turn transfer its phosphoryl group to either Ssk1 in the cytoplasm or Skn7 in the nucleus (Ketela et al., 1998). In addition to Skn7, Ypd1 may also interact with other proteins in the nucleus. One example is Apn1, an AP endonuclease in the base-excision repair pathway that is crucial for fixing damages caused by alkylating agents such as MMS (Krogan et al., 2006). It is possible that MMS treatment could impact the phosphorelay between Ypd1 and Ssk1 by enhancing the interaction between Ypd1 and its interacting partners in the nucleus such as Apn1 or Skn7, thus reducing the availability of cytoplasmic phosphor-Ypd1 to bind and transfer its phosphoryl group to Ssk1. In addition to relaying the phosphoryl group to Ssk1, another established function of Ypd1 is binding to phosphorylated Ssk1 and shielding Ssk1 from the action of phosphatases that dephosphorylate Ssk1 (Saito and Posas, 2012; Dexter et al., 2015). Thus, diminished interaction between Ypd1 and Ssk1 could also contribute to a decrease in the phosphorylation level in Ssk1.

As a stress-activated protein kinase, Hog1 can be activated by a large variety of stressors such as hyperosmolarity, heat, cold, citric, and acetic acid, oxidative stress, methylglyoxal, bacterial lipopolysaccharide, glucose starvation, curcumin, arsenite/arsenate, and cadmium (Lee et al., 2019). While it is well-established how hyperosmolarity and arsenite/arsenate activate Hog1, the mechanisms by which Hog1 is activated by other stressors are less clear. With the impairment of Ypd1/Ssk1 phosphorelay as a new mechanism for Hog1 activation, in the future it would be interesting to examine if it is utilized by other stressors to activate Hog1.

Our study indicates that one function of Hog1 activation by MMS is to stimulate autophagy, as the level of MMS-induced autophagy is significantly decreased when Hog1 is not present. How does Hog1 stimulate autophagy? Given that MMS-induced Hog1 is predominantly located in the cytoplasm, the relevant target should be a cytoplasmic protein. An early study indicated that cytoplasmically activated Hog1 can stabilize Atg8 in response to ER stress (Bicknell et al., 2010). Atg8 has a role in determining the size of autophagosome (Backues et al., 2012), thus stabilizing Atg8 would increase the level of the protein, which in turn would increase autophagosome size and therefore upregulate autophagy. It would be interesting to examine whether Hog1 also regulates Atg8 stability in a genotoxic stress-dependent manner. In addition, because elevation of autophagy by genotoxic stress is a highly conserved phenomenon (Eliopoulos et al., 2016) and the Hog1 homolog p38 is known to be activated by genotoxic stress (Zlotorynski, 2018), the possibility that p38 activation contributes to the regulation of autophagy is definitely worth pursuing.

In summary, our study clearly indicates that MMS activates a mitogen-activated protein kinase Hog1 to regulate autophagy, and activation of Hog1 is potentially achieved via disrupting the phosphorelay between its two upstream components Ypd1 and Ssk1. Given the highly conserved nature of mitogen activated protein kinases and autophagy, our study sheds new light on how cells respond to genotoxic stressors in general and MMS in particular.

## Materials and Methods

### Strains and Plasmids

Standard methods for the growth, maintenance, and transformation of yeast and bacteria and for the manipulation of DNA were used throughout. The yeast *S. cerevisiae* strains used in this study are BY4741 (*MAT****a*** *leu2*Δ *met15*Δ *his3*Δ *ura3*Δ), BY4741-derived mutants lacking *PTC1, SHO1, MSB2, OPY2, STE20, STE11, SSK1, SSK2, SSK22, PBS2, HOG1* (Research Genetics, Huntsville, AL), BY4741-derived strains with a TAP tag on the genomic locus of *YPD1, SSK1, SSK2*, and *PBS2* (Ghaemmaghami et al., 2003), and BY4741-derived strain with a GFP tag on the genomic locus of *HOG1* (Huh et al., 2003). The *ssk2*Δ *ssk22*Δ mutant was created by replacing *SSK2* open reading frame with a *URA3* marker in the *ssk22*Δ mutant; the *ssk2*Δ *ste11*Δ mutant was created by replacing *SSK2* open reading frame with a *URA3* marker in the *ste11*Δ mutant; the *ssk1*Δ *sho1*Δ was created by replacing *SSK1* open reading frame with a *URA3* marker in the *sho1*Δ mutant. BY4741-derived mutant carrying a catalytic inactive allele of *HOG1* (hog1^K52R^) was generously provided by Dr. Stephen Parnell at University of Kansas. The expression plasmid 416-*GFP-ATG8* was generously provided by Dr. D. Klionsky at University of Michigan, and the *STL1*-lacZ reporter construct was generously provided by Dr. Francesc Posas at Universitat Pompeu Fabra.

### Phosphorylation and Immunoblotting Bioassays

Hog1 phosphorylation was analyzed using antibodies that recognize phosphorylated and thus activated Hog1. For all the immunoblotting analysis, mid-log phase cells were grown on appropriate medium, treated or not treated with 0.08% MMS or other conditions as indicated. Proteins were extracted via trichloroacetic precipitation, following procedures described previously (Slessareva et al., 2006). Whole cell extracts were resuspended in boiling SDS-PAGE sample buffer (62.5 mM Tris-HCl, pH 6.8, 10% glycerol, 2% SDS, 1% 2-mercaptoethanol, 0.0005% bromphenol blue) for 5 min. Following either regular SDS-PAGE or phos-tag (10 μM, from Wako) SDS-PAGE and transfer to nitrocellulose, the membrane was probed with antibodies to phosphor-p38 at 1:1000 (from Cell Signaling Technology), Hog1 at 1:500 (from Santa Cruz), GFP at 1:5,000 (from Abcam, ab13970), and Protein A at 1:10,000 (from Sigma). Immunoreactive species were visualized by enhanced chemiluminescence detection (Pierce) of horseradish peroxidase-conjugated anti-rabbit IgG (Bio-Rad), anti-mouse IgG (Santa Cruz), or anti-Chicken IgY (Abcam). Specificity of detection was established using cell extracts without tagged proteins as negative controls. All experiments have been repeated at least three times. Immunoblotting signals were quantified with ImageJ software, and the dot bar graphs were generated using Interactive Dotplot (Weissgerber et al., 2017), and the bars represent standard deviations. Where indicated, the data were statistically analyzed by *t*-test, with *p* < 0.050 considered significant.

### Microscopy Analysis

Cells expressing GFP-tagged Hog1 were grown to either mid-log phase or treated with nitrogen starvation condition for the indicated times. Cells were concentrated and 10 μl of concentrated cell suspensions were placed on a slide with a thin-layer of 0.5% agar and visualized by fluorescence microscopy using an Olympus FV1000 laser scanning confocal microscope. Fluorescence images were analyzed and quantified with ImageJ software.

## Data Availability Statement

The original contributions presented in the study are included in the article/supplementary materials, further inquiries can be directed to the corresponding author/s.

## Author Contributions

SH performed most experiments. FW and DZ contributed to strain creation and experiments. YW and SH designed the experiments and wrote the manuscript. All authors contributed to the article and approved the submitted version.

## Conflict of Interest

The authors declare that the research was conducted in the absence of any commercial or financial relationships that could be construed as a potential conflict of interest.
